# Next-generation sequencing-based genomic profiling analysis reveals novel mutations for clinical diagnosis in Chinese primary epithelial ovarian cancer patients

**DOI:** 10.1186/s13048-019-0494-4

**Published:** 2019-02-20

**Authors:** Lei Zhang, Min Luo, Hongying Yang, Shaoyan Zhu, Xianliang Cheng, Chen Qing

**Affiliations:** 1 School of Pharmaceutical Sciences & Yunnan Key Laboratory of Pharmacology for Natural Products, Kunming Medical University, 1168 Western Chunrong Road, Yuhua Street, Cheng Gong District, Kunming, Yunnan 650500 People’s Republic of China; 2 Department of Gynecology, Yunnan Tumor Hospital & The Third Affiliated Hospital of Kunming Medical University, 519 Kunzhou Road, Xishan District, Kunming, Yunnan 650118 People’s Republic of China

**Keywords:** Epithelial ovarian cancer, Oncogenesis, Expression profiling, Pathway analysis

## Abstract

**Background:**

Ovarian cancer (OC) is one of the most malignant gynecological tumors, associated with excess death rate (50–60%) in ovarian cancer patients. Particularly, among newly occurred ovarian cancer patients, 70% of clinical cases are diagnosed at the advanced stage, which definitely delay the timely treatment and lead to high mortality rate within 5 years post diagnosis. Therefore, identification of sensitive gene markers, as well as development of reliable genetic diagnosis, are important for the early detection and precise therapy for OC patients. This study aims to identify novel genetic mutations and develop a feasible clinical approach for early OC diagnosis.

**Methods:**

The OC tissue-derived DNA sample was acquired from 31 OC patients, and the somatic gene mutations will be identified after comparison with normal samples, using Genome-wide analysis and next-generation sequencing.

**Results:**

A total of 463 somatic mutations, which were considered as potential pathogenic sites, were assigned to 473 genes. Among them, 15 genes (TP53, TTN, MUC16, OR4N2, BRCA1, CAD, CCDC129, INSR, NAV3, NELL2, NRAS, OBSCN, PGLYRP4, RBM15B and TRPC7) were mutated on at least two sites. These genes were mapped to RNA sequencing (RNAseq) data, and a total of 117 genes had an absolute fold- change ≥ 2 and *p* ≤ 0.01. Five genes were mutated in at least two OC patients. Gene ontology (GO) classification indicated that a majority of genes participated in biological processes. Kyoto Enrichment of Genes and Genomes (KEGG) enrichment pathway analysis revealed that the genes were mainly involved in the regulation of metabolic signaling pathways.

**Conclusions:**

Taken together, this study identified several novel genetic alterations pathway for early clinical diagnosis and provided abundant information for understanding molecular mechanisms of the OC occurrence and development.

**Electronic supplementary material:**

The online version of this article (10.1186/s13048-019-0494-4) contains supplementary material, which is available to authorized users.

## Background

Ovarian cancer (OC) is one of the most malignant gynecological tumors worldwide [1]. Although abdominal pain, abdominal distension, gastrointestinal discomfort, and irregular menstruation appear in the initial stage of OC [2, 3], these signs and symptoms are relatively non-specific and difficult to distinguish from other diseases. In addition, the deep anatomy location of the ovary in the pelvic cavity and the absence of effective screening tools prevent the detection of this disease at early - stage. Indeed, among newly diagnosed OC cases, 70% of patients are found at stage III and IV OC (Federation of Gynecology and Obstetrics (FIGO), III-IV period) [1, 4, 5], directly leading to the failure in timely treatment. Therefore, OC is considered as a “silent killer.” Despite the inhibition of tumor growth can be achieved using initial aggressive treatment, both high recurrence rate (70%) and extremely poor overall survival (OS) rate are still observed in patients [6], and the average 5-year OS rate for all stages is 45.6% [1, 4–9]. According to reports from the National Cancer Institute (NCI), about 140,000 people die each year from OC worldwide [10]. For the past 10 years in China, the incidence and mortality of OC have increased by 30 and 18%, respectively evidenced by ~ 15,000 deaths yearly [11].

Epithelial ovarian cancer (EOC) is the predominant type (about 90%) of OC, and categorized into four types according to histological features: serous, mucinous, endometrioid, and clear cell [12]. Recently, EOC has also been considered as two broad, simplified groups: type I including low-grade serous, endometrioid, clear cell, mucinous, and transitional cell carcinomas, and type II consisting of high-grade serous carcinomas, undifferentiated carcinomas, and carcinosarcomas [12, 13]. Particularly, EOC contributes to 50–70% of primary ovarian tumors and 85–90% of ovarian malignant tumors in China [11]. Due to the genetic heterogeneity of EOC with different pathological characteristics and molecular genotypes, there is a desperate and urgent demand for figuring out the molecular pathogenesis of EOC and identifying novel therapeutic targets and biomarkers, allowing “precision medicine” to be achieved in clinical practice.

Recently, microarray technologies have been used to elucidate the complexity of genomic alterations of OC and identify biomarkers and potential therapeutic targets for developing comparative medicine model [14–18]. A 126-gene expression profile analysis has been performed on primary tumor tissues and successfully employed as genetic signature to predict OS in high-grade serous OC [19]. Accordingly, a recent study also demonstrated the correlation between BRCAness profile and the survival of EOC patients [20]. Somatic mutations of ARID1A (the AT rich interactive domain 1A (SWI-like)) [21] and PIK3CA (phosphatidylinositol-4, 5-bisphosphate 3-kinase, catalytic subunit alpha) genes [22] have been frequently detected in EOC, which was further supported by the emergence of 45 somatic mutations in 34 genes, including PIK3CA and ARID1A mutations in the most independent Ovarian clear cell carcinoma (OCCCs) [18]. It has been reported that plasma metabolites can be utilized to predict OS and show difference between short-term mortality and long-term survival in EOC patients [16].In addition to tumor biopsy samples, circulating cell-free DNA (cf-DNA) and circulating tumor cells (CTCs) [23] have emerged as “liquid biopsies” for non-invasive biomarkers in both the early and advanced diagnosis, prognosis, and in the identification of resistance mutations in OC.

In the past 5 years, the next-generation sequencing (NGS) technology has become widely available to determine a patient’s precise genetic profiling and identify novel mutations for new drug targets and individualized treatment schemes.

This study identified novel differentially expressed gene (DEG) mutations through comparing the gene expression profiles between EOC and normal healthy tissues from 31 EOC patients in Yunnan Province of China along with the analysis of the Gene Ontology (GO) functions and pathways of the candidate genes involved in EOC progression. The results of the present study provide the better understanding of the molecular mechanisms of EOC as well as potential biomarkers for prognosis and effective therapeutic targets.

## Results

### Clinico-pathologcic patient characteristics

There were 13 unilateral and 28 bilateral ovarian tumors among all 31 enrollment cases (age range, 45–75 years; median age, 55). Particularly, 3 cases were diagnosed with clear cell adenocarcinoma, 22 cases with serous adenocarcinoma, 3 cases with endometrioid adenocarcinoma, 1 case with mucinous adenocarcinoma, and 2 cases with undifferentiated carcinoma.

We used the international clinical staging system, which is established by the Federation of Gynecology and Obstetrics (FIGO), to determine cancer stage. Seven cases were diagnosed at stage I (22.58%), 4 cases at stage II (12.90%), 18 cases at stage III (58.07%), and 2 cases at stage IV (6.45%) (Table 1).

**Table 1:** Clinico-pathologic characteristics of enrolled 31 cases

Characteristics	Data
All cases	31
Median age (range)	55 (45–75)
Site of primary tumor	
Single/bilateral	13/18
Single: right/left	9/4
Histological type	
Serous adenocarcinoma	22
Low-grade/ High-grade	3/19
Mucinous adenocarcinoma	1
Endometrioid adenocarcinoma	3
G1/G2/G3	0/1/2
Clear cell adenocarcinoma	3
Undifferentiated carcinoma	2
FIGO staging	
I	7
II	4
III	18
IV	2
Prognosis	
Survival	24
Death	7

*G1* well-differentiated, *G2* moderately differentiated, *G3* poorly differentiated

### Characteristics of somatic mutations in 31 Chinese EOCs

A total of 1598 somatic SNVs (single nucleotide variants) were replied from the raw NGS data in 31 EOC patients, among which one synonymous mutation in SPRR3 on chromosome 1 was detected in 4 patients with a mutation of allele C to T, and four nonsynonymous mutations were simultaneously appeared in 2 patients (Table 2). The mutations were confirmed by Sanger sequencing (Additional file 1: Figure S1). Three SNVs, including KRTAP4–3, FBXW10 and ZNF814, were present in the gene polymorphism database; however, position 7,577,539 of TP53 was mutated from G to A in 2 patients, but was not detected in the database. All SNVs were then filtered according to the following premises: 1) synonymous mutations, 2) known minor allele frequency (MAF) > 1% in 1000 Genomes and ExAc databases, and 3) introns, intergenic, and UTR5 sites. Consequently, a total of 463 somatic mutations (Additional file 2: Figure S2A), which were considered as potential pathogenic sites, were assigned to 437 genes.

**Table 2:** A list of the most common mutation sites in 31 EOCs

Gene	CHR	POS	Ref	Alt	Count	rsID
FBXW10	chr17	18,682,505	T	C	2	rs1024657
KRTAP4–3	chr17	39,324,333	T	A	2	rs12953139
TP53	chr17	7,577,539	G	A	2	na
ZNF814	chr19	58,385,748	G	A	2	rs145250945

*CHR* chromosome, *POS* position of the mutation, *Ref* reference base, *Alt* alteration base, *Count* number of patients sharing a mutation, *rsID* (reference SNP) - number of known mutations in dbSNP database

The average number of somatic mutations identified in each gene was 1.06, ranging from 1 to 9; 15 genes were altered at least twice (Table 3), which resulted in 9 mutations in TP53 (Tumor protein p53), 4 in TTN (Titin), 3 in MUC16 (Mucin 16), and 2 mutation sites in the following genes: BRCA1 (Breast cancer 1), CAD (Carbamoyl-Phosphate Synthetase 2, Aspartate Transcarbamylase, and Dihydroorotase), CCDC129 (Coiled-coil domain containing 129), INSRR (Insulin Receptor Related Receptor), NAV3 (Neuron navigator 3), NELL2 (Neural EGFL like 2), NRAS (NRAS Proto-Oncogene, GTPase), OBSCN (Obscurin, Cytoskeletal Calmodulin And Titin-Interacting RhoGEF), PGLYRP4 (Peptidoglycan recognition protein 4), RBM15B (RNA Binding Motif Protein 15B), and TRPC7 (Transient Receptor Potential Cation Channel Subfamily C Member 7). Particularly, somatic mutations within the tumor suppressor gene TP53 accounted for the highest frequency of alterations detected in the present study.

**Table 3:** Summary of 15 genes mutated at least twice

Gene Name	Chromosome	Count gene	Cosmic-ID
TP53	17	9	9
TTN	2	4	0
MUC16	19	3	1
INSRR	1	2	0
NRAS	1	2	2
OBSCN	1	2	0
PGLYRP4	1	2	0
CAD	2	2	0
RBM15B	3	2	0
TRPC7	5	2	0
CCDC129	7	2	0
NAV3	12	2	1
NELL2	12	2	0
OR4N2	14	2	0
BRCA1	17	2	1

*Count* number of mutations in each gene, *Cosmic-ID* number of mutations in cosmic database

### Identification of significant genes with RNA-Seq analysis

Four hundred thirty-seven genes were mapped to RNA-Seq data to analyze and compare the RNA expression values between EOC and normal tissue samples, and there were total of 117 genes detected with an absolute fold-change ≥2 and *p* ≤ 0.01 as the cut-off criteria (Additional file 2: Figure S2B). Among them, five genes were mutated in at least two EOC cases (Table 4): 4 in TTN, 3 in MUC16, and 2 mutations in the following genes: BRCA1, INSRR, or NRAS. Such mutations may be correlated with clinical benefit of targeted therapies. For instance, BRCA1 mutations were potentially sensitive to PARP (poly ADP ribose polymerase) inhibitors and DNA damaging agents. The mRNA expression pattern was consisitent with the Immunohistochemistry (Additional file 3: Figure S3).

**Table 4:** Five top genes mutated at least twice with an absolute fold-change ≥ 2 and *p* ≤ 0.01 of RNA expression between tumor and normal tissue samples

Gene information			DNA mutant	RNA-Seq	
Gene	NAME	CHR	Count	Fold-change	*p-*value
TTN	Titin	2	4	−2.48	0.0054772
MUC16	Mucin 16, cell surface associated	19	3	8.05	0.00013
BRCA1	Breast cancer1, early onset	17	2	2.37	0.0011747
NRAS	Neurablastoma RAS viral oncogene homolog	1	2	2.21	0.000907
INSR	Insulin receptor	1	2	−2.08	0.0011705

### GO terms of 117 genes

**Fig. 1:**
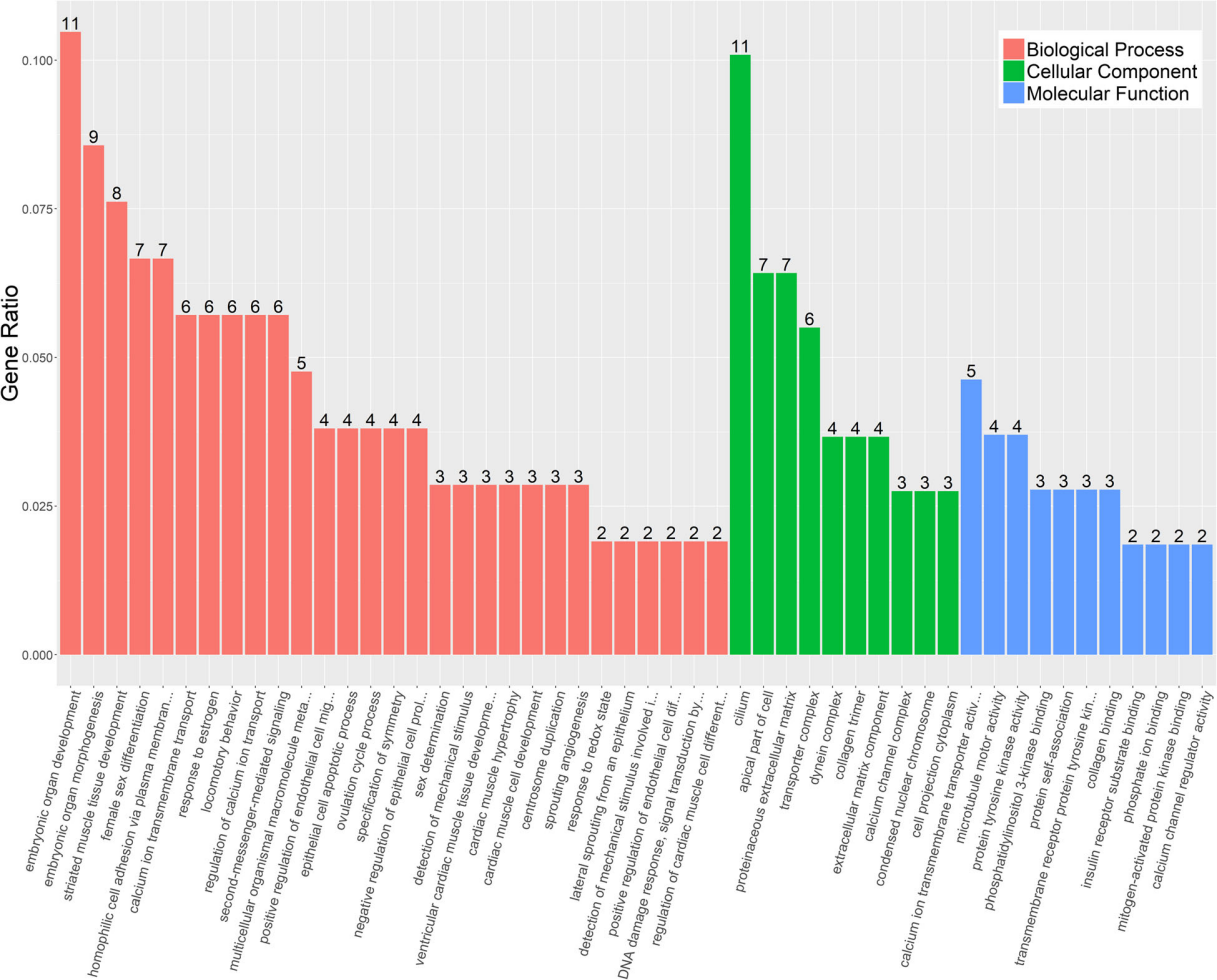
GO clustering analysis. Annotations are grouped by biological process, cellular component and molecular function based on the GO annotation information

GO classification of 117 genes was categorized into three groups, which are involved in the regulation of biological process (BP), cellular component (CC), and molecular function (MF), respectively (Fig. 1). Among them, numerous BP-regulated genes were matched compared to the other two categories. The two most abundant GO BP functional groups - “embryonic organ development” and “embryonic organ morphogenesis” - are not surprising because these genes are involved in embryonic organ development to perform a specific function(s). Among CC, “cilium” was the largest enriched category, followed by “apical part of cell” and “proteinaceous extracellular matrix”. In the MF categories, slightly more genes were enriched in the GO term “calcium ion transmembrane transporter activity”, followed by “microtubule motor activity” and “protein tyrosine kinase activity”. The corresponding genes of these significant GO terms, therefore, might play important roles in EOC.

### KEGG enrichment pathways of 117 genes

**Fig. 2:**
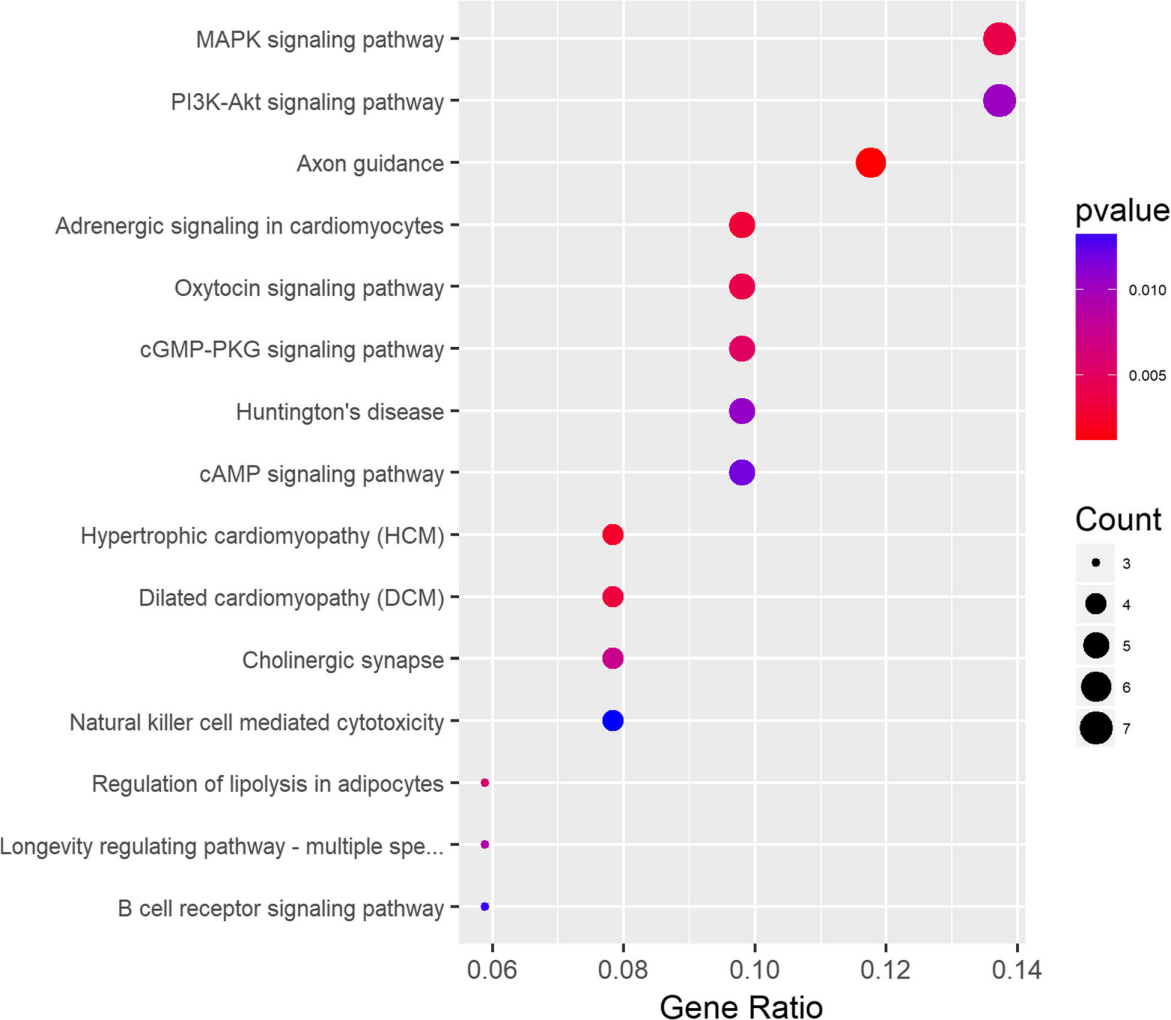
Scatterplot of enriched KEGG pathways for DEGs (differentially expressed genes) between case and normal tissue samples. Gene ratio Rich factor is the ratio of the DEG (differentially expressed gene) number to the total gene number in a certain pathway

To better understand potential underlying mechanisms in EOC, we performed KEGG pathway analyses using 117 genes with an absolute fold-change value above 2, while *p* value is less than 0.01, and identified several molecular mechanisms that regulate pathogenesis of EOC (Fig. 2). Genes were predominantly enriched in pathways in the mitogen-activated protein kinase (MAPK) and PI3K-Akt signaling pathways, which are followed by axon guidance, adrenergic signaling in cardiomyocytes, oxytocin signaling and cGMP-PKG signaling.

### Reactome enrichment pathway of 117 genes

**Fig. 3:**
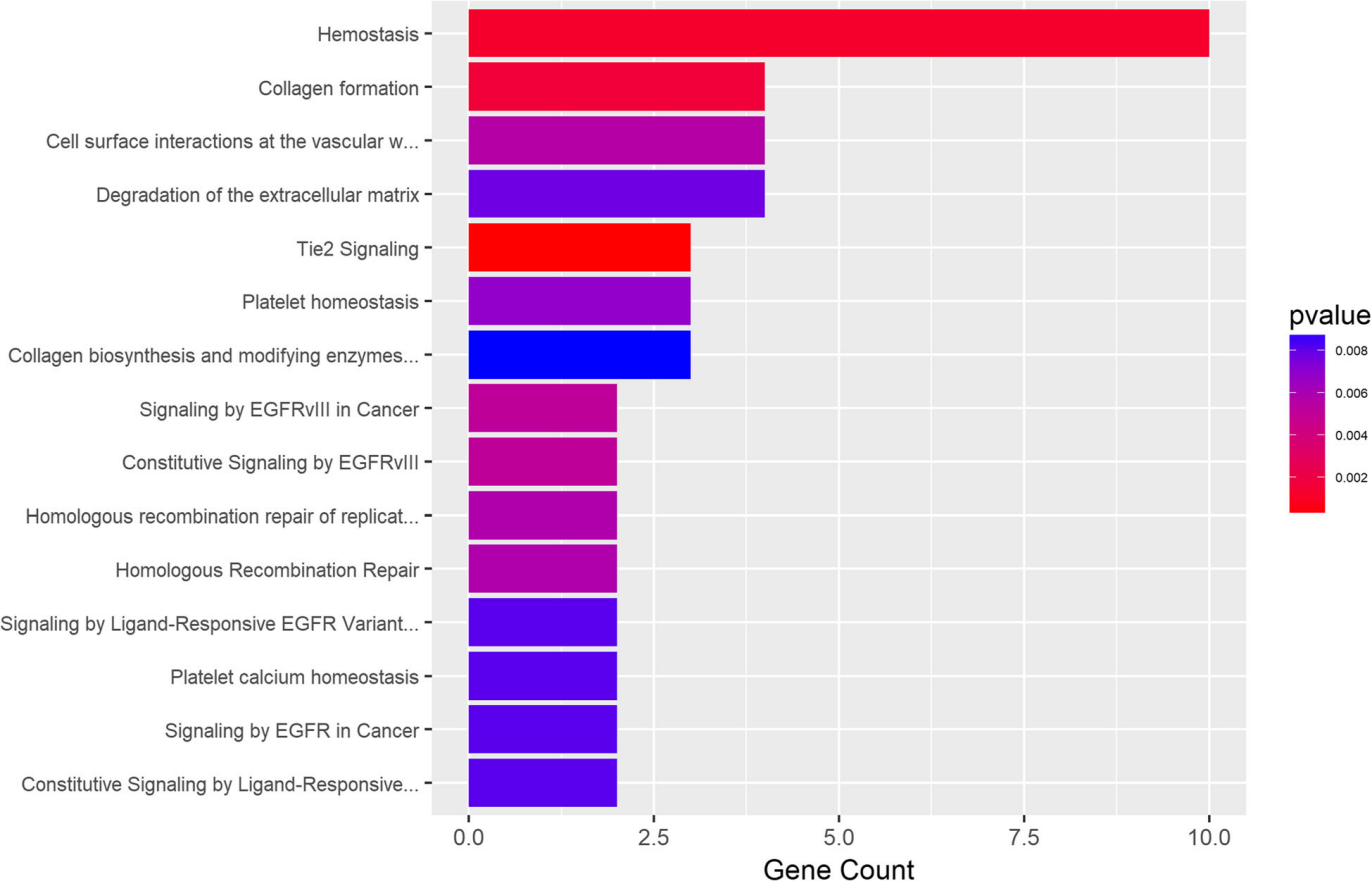
Enriched Reactome pathways. The results for the ‘overrepresentation analysis’, presented as a list of clickable links of enriched events. The warmer the color, the higher the level of overrepresentation in the given pathway

As shown in Fig. 3, the Reactome enrichment pathway analysis revealed a set of genetic pathways involved in the regulation of hemostasis, collagen formation, and Tie2 signaling, which are biological cellular and molecular events post the EGFRvlll-induced the interaction between cancer cell surface and vascular wall, and these processes have been proven to be significantly associated with EOC. Hemostasis is involved in the growth and spread of malignant tumors, while collagen formation acts as a double-edged sword in tumor progression. Accompanying losses of tumor suppressor genes (p53), mutant oncogenes facilitate expression of angiogenic and pro-inflammatory factors, as well as change the cancer cell coagulum, including the levels of tissue factor and other mediators.

## Discussion

The present study is the preliminary research of the complexity of genomic alterations in EOC based on NGS. Particularly, the excessive mutation frequency was identified in TP53 gene, which is consistent with previous reports [16, 18]. Ross et al. (2013) identified TP53 mutations in 79% of OC patients [16], which were more common in papillary serous carcinomas (83%) than in non-papillary serous tumors (50%). Maru et al. (2016) reported multiple TP53 mutations in at least two independent OCCC samples [18]. In agreement with the results of Ross et al. (2013), in which 8 BRCA1 mutations were reported in 44 EOC samples [16], we also identified at least two BRCA1 mutations in the present study. BRCA1 or TP53-gene mutation are predisposed for the increased susceptibility to a variety of cancers, including EOC, and both tumor suppressor genes have been implicated in DNA damage response pathways. Although function in separate pathways to suppress tumorigenesis, BRCA1 can serve as a coactivator to physically interact with TP53 [24, 25]. TP53-encoding gene is positioned the short arm of chromosome 17 [26] and responsible for the expression of the tumor suppressor protein p53 [27] in multicellular organisms. Especially, p53 plays a critical role in determining the repair of the damaged or mutated DNA. The NGS results in the present study confirmed a high degree of TP53 mutations, which is in agreement with previous NGS and further supported by the DNA sequencing, and DNA copy number assessments, as well as mRNA, microRNA and DNA promoter methylation status assays [16, 18, 28, 29]. The tumor suppressor gene, BRCA1, is positioned at the long arm of chromosome 17 [30] and responsible for the expression of breast cancer type 1 susceptibility protein, a functional component of BRCA1-associated genome surveillance complex (BASC) [31]. BASC plays a role in gene transcription, repair of DNA double-strand breaks, protein ubiquitination, and post-transcriptional regulation. Somatic BRCA1 mutations have been identified as a significant feature of high grade serous ovarian carcinoma [32]. Loss of BRCA1 activity, either by germ-line mutations or by down-regulation of gene expression, makes DNA damaging susceptible to therapeutic chemical agents, including platinum, or possibly agents that inhibit DNA repair pathways (i.e. poly ADP ribose polymerase inhibitors), increasing the risk of developing OC by 55% [16, 32]. The beyond molecular mechanisms, through which alternations in TP53 and BRCA1 sensitize cancer cells to cytotoxic or targeted- therapies, remain unclear. However, more detailed understanding of aberrant gene expression could eventually produce beneficial effects on cancer therapy.

The additional mutated genes - TTN, MUC16, OR4N2, CAD, CCDC129, INSR, NAV3, NELL2, NRAS, OBSCN, PGLYRP4, RBM15B and TRPC7 - have been previously reported, which revealed the highly association with cancer development [33]. Among these oncogenic genes, MUC16 encodes a repeating peptide epitope of mucin [34] that promotes cancer cell proliferation and inhibits anti-cancer immune responses [33–35]. MUC16 is well-known as a clinically reliable diagnostic and therapeutic marker of EOC and is served as differentially diagnose pelvic masse [35]. MUC16 participates in the several signaling pathways, including defective C1GALT1C1, which causes Tn polyagglutination syndrome (TNPS) and CLEC7A (Dectin-1) signaling. It noteworthy that somatic mutations of other genes have not been reported in EOCs cases. TTN and CAD were identified as mutated oncogenes; TTN ranks at the top of 518 potential protein kinase cancer genes [33] and it encodes the largest polypeptide that [36] is expressed in many functional cell types in oncogenesis [37]. INSR encodes a protein that acts as an extracellular pH sensor in the regulation of acid-base balance in humans. INSR expression shows a correlation with degree of apoptosis and dedifferentiation in human neuroblastomas, and is co-expressed with insulin-like growth factor 1 receptor [38]. CAD encodes a trifunctional protein, which is regulated by the MAPK cascade [39]. NAV3 and OBSCN-encoding genes show a highly mutant frequency in human tumors. NAV3 encodes the protein, which contain both-coiled-coil domains and a conserved AAA domain, a featured ATPases functional motif regulating a variety of cellular activities including epithelial migration and invasion [40]. OBSCN has been implicated in cancer biogenesis and development through regulating cell survival [41]. NRAS is a member of the Ras family oncogenes and encodes the GTPase proteins that activate more than 20 signaling pathways, and these signaling cascades are involved in the regulation of essential cellular functions, such as proliferation, survival, and migration, cell division, cell differentiation, and the self-destruction of cells (apoptosis). Upon mutation, oncogenes can convert normal cells into cancerous cells. NRAS mutations were reported to function as reliable predictors of resistance to anti-EGFR (epidermal growth factor receptor) monoclonal antibody therapy [42]. Thus, further analysis is needed to identify the functional role of these mutations in EOC carcinogenesis to identify new prognostic biomarkers and develop novel therapeutic targets. However, it is important to remark that the clinico-pathological features of EOCs and the prognostic impact of these oncogenic mutations remains unclear.

This work presents a comprehensive gene expression profile of EOC. In the present study, NGS was used to assess differential gene expression between EOC and healthy samples, and the further craft of a pathway signature of EOC was performed using GO and KEGG enrichment analyses. A majority of the 117 candidate genes were classified into “pathways in cancer” and “axon guidance”, upon the activation of “cGMP-PKG signaling pathway”, “oxytocin signaling pathway”, and “adrenergic signaling in cardiomyocytes”. Some of the common pathways were identified as significant in both KEGG and Reactome analyses. “Pathways in cancer” is a comprehensive pathway of multiple cellular processes and crosstalk during cancer development, including the p53 signaling pathway and MAPK signaling pathway. Alternations in TP53 gene expression occur in more than 50% of human tumors [27, 28], leading to the severe reduction of tumor suppression and increasing the possibility that a cell will perform the uncontrolled division. Axon guidance is a process by which axons stretch to their correct targets, and axon guidance pathway genes have been implicated in cancer cell growth, survival, invasion, and angiogenesis [43]. Unfortunately, the incidence of aberrations in these genes in cancer is largely unknown. Several axon guidance genes, including TP53, have been implicated in human cancers including pancreatic carcinogenesis [43]. The cGMP/PKG signaling pathway is involved in cell cycle progression, cellular proliferation, and chromosomal instability [44], playing as an important role in regulating the proliferation and survival of human renal carcinoma cells. In tumors, oxytocin acts as a growth regulator via the activation of the oxytocin receptor, a specific G-coupled transmembrane protein. Adrenergic signaling has been found to regulate multiple cellular processes that contribute to the initiation and progression of cancer, including inflammation, angiogenesis, apoptosis/anoikis, cell motility and trafficking, activation of tumor-associated viruses, DNA damage repair, cellular immune response, and epithelial–mesenchymal transition.

Recent studies have revealed that both pathway signature and mutation signature are important to delineate carcinogenesis in individual cancers [18, 33]. The current study used NGS to deep-sequence hundreds of cancer-related genes from clinical grade tumor samples and elucidated an unexpectedly high frequency of actionable genomic events that may inform targeted treatment decisions. Further work includes the selection and validation of individual genes as biomarkers in a well-designed research with appropriate molecular diagnostic gating to promote precision therapies that specifically target molecules of tumor-driving signaling pathways and predict the response of an individual patient on such a targeted therapy.

## Conclusions

In conclusion, our research identified several novel genetic alterations and pathway of OC by NGS, which provided abundant information for understanding molecular mechanisms of the OC, and even contributed to early clinical diagnosis. However, further studies with selective candidate genes and larger sample size are needed to define their role in occurrence and development of OC.

## Methods

### Patients and pathological materials

Thirty-one patients, who enrolled in the present study, were pathologically diagnosed with EOC at the Gynecology Division of Yunnan Tumor Hospital during February to September, 2016 (Table 1). Patients with co-occurrence of other malignancies or having received radiotherapy, chemotherapy, or other anti-cancer treatments were excluded. Pathological data of all patients were appropriately recorded, which allow track treatment and survival results to be followed up.

### Quality assessment of DNA and RNA

All patients were subject to fast for at least 8 h before surgery, and blood samples (10 mL) were drawn into EDTA-K2 anti-coagulative tubes, from which white blood cells were isolated to extract genomic DNA using QIAamp DNA Kits (Qiagen,Cat. No. 51104). In the single tumors, fresh tissues of ovarian cancer and cortex from the ovarian on the other side were sampled during the operation. The ovarian cortex (or ovarian surface epithelium, OSE) was use as control group, if the tissue was confirmed as normal by postoperative pathological examination. DNA and RNA were isolated by the ALLPrep DNA / RNA Mini Kit (Qiagen, CatNo.80204). In the presence of bilateral tumors, no normal ovarian tissues were sampled, so leukocyte DNA was used as a normal control. The purification and quantification of DNA and RNA were measured using a Nano-Drop ND-8000 Spectrophotometer (Thermo Fisher Scientific, Waltham, MA, USA). DNA and RNA integrity were determined by 1% agarose gel electrophoresis.

### NGS library preparations

The NGS library was constructed using the SeqCap EZ System from NimbleGen (Arrowhead Madison, Inc. Madison, WN, USA) according to the manufacturer’s instructions. Briefly, genomic DNA was sheared to size the target sequences to roughly 300 base pairs that were converted to double-stranded DNA. The cDNA was then end repaired and specific oligonucleotide adapter ligated for multiplexing index and ligation-mediated polymerase chain reaction (LM-PCR), followed by incubation with SeqCap biotinylated DNA baits, and purification of the hybrids using streptavidin-coated magnetic beads. After amplification with a maximum of 18 cycles, the libraries were sequenced using 100-bp pair-ended reads on the Illumina HiSeq 3000 platform (Illumina, Inc., San Diego, CA, USA).

Libraries for RNA-Seq were prepared using a KAPA Stranded RNA-Seq Kit. The workflow consisted of mRNA enrichment, cDNA generation, and end repair to generate blunt ends, A-tailing, adaptor ligation, and PCR amplification. Different adaptors were used for multiplexing samples in one lane. Sequencing was performed on an Illumina HiSeq 3000 for a single read 50 runs. Data quality check was done on an Illumina SAV. De-multiplexing was performed with the Illumina Bcl2fastq2 v 2.17 program.

### Sequencing and mutation analysis

The raw sequence data were aligned to the GRCh37 human reference genome using BWA v0.7.7-r411 (http://bio-bwa.sourceforge.net/). PCR duplicates were marked using the Mark Duplicates program in Picard-tools-1.115 tool set (https://github.com/broadinstitute/picard). The DepthOfCoverage functionality of Genome Analysis Tool Kit (GATK-v3.2–2) (https://software.broadinstitute.org/gatk/) was used to identify INDELs (insertions and deletions). Alignment coverage was calculated using bedtools (http://bedtools.readthedocs.io/en/latest/). Samtools (http://samtools.sourceforge.net/) was used to call the SNVs (single nucleotide variants) and small INDELs. Varcscan2 (http://varscan.sourceforge.net/) was used to call somatic SNV. All SNVs were functionally annotated using the Annovar program (http://www.openbioinformatics.org/annovar/). The median sequencing depth of these samples is more than 50 (Additional file 4: Figure S4).

RNA-Seq reads were first mapped to the latest UCSC transcript set using Bowtie2 version 2.1.0 and the gene expression level was estimated using RSEM v1.2.15. TPM (transcript per million) was used to normalize gene expression. The comparison was performed between each pair of tumor and normal tissues of the same patient. Differential expression analysis was performed by the paired test in R package edgeR, which adjusted for any differences between the patients. DEGs were identified using the edgeR program (http://bioconductor.org/packages/release/bioc/html/edgeR.html). Genes showing altered expression with an absolute fold-change ≥ 2 and *p* ≤ 0.01 were considered DEGs. Fold change was calculated by the predFC function in edgeR package (https://www.rdocumentation.org/packages/edgeR/versions/3.14.0/topics/predFC).

### GO and KEGG analysis

Pathway and network analyses were performed using Ingenuity (IPA). IPA computes a score for each network according to the fit of the set of supplied focus genes. These scores indicate the likelihood of focus genes to belong to a network versus those obtained by chance. A score > 2 indicates a 99% confidence that a focus gene network was not generated by chance alone. The canonical pathways generated by IPA were the most significant for the uploaded data set. Fischer’s exact test with false discovery rate (FDR) option was used to calculate the significance of the canonical pathway.

GO analysis is commonly used to annotate genes and their products, whereas the KEGG pathway database is used to identify functional and metabolic pathways. We used the Database for Annotation, Visualization and Integrated Discovery (DAVID) database to perform GO and KEGG functional enrichment analyses for DEGs. *p* < 0.05 was considered statistically significant.

## Additional files

Additional file 1: **Figure S1.** Result of sanger sequencing. (A) Eleven mutations which were sanger sequenced. (B) - (H) Seven mutations which were validated to be true. (PDF 83 kb)

Additional file 2: **Figure S2.** Distribution of genes with novel mutations based on individual patients. (A) Distribution of 463 novel mutations in each patient with cancer. (B) Distribution of the 117 genes with novel mutations based on individual patients. (PDF 4100 kb)

Additional file 3: **Figure S3.** Representative immunohistochemical staining for EPS8L1 expression. (A/B): Negative expression of EPS8L1 in normal ovarian tissue, 200×/400×. (C/D) Weakly positive expression of EPS8L1 in EOC tissue, 200×/400×. (E/F) Positive expression of EPS8L1 in EOC tissue, 200×/400×. (G/H) Strongly positive expression of EPS8L1 in EOC, 200×/400 ×. (JPG 303 kb)

Additional file 4: **Figure S4.** Target region coverage. (A) Target region coverage of normal control group in sample 1–10. (B) Target region coverage of tumor group in sample 1–10. (C) Target region coverage of normal control group in sample 11–20. (D) Target region coverage of tumor group in sample 11–20. (E) Target region coverage of normal control group in sample 21–31. (F) Target region coverage of tumor group in sample 21–31. (PDF 632 kb)

## Abbreviations

cf-DNA: cell-free DNA
CTCs: Circulating tumor cells
DEG: differentially expressed gene
EOC: Epithelial ovarian cancer
FIGO: Federation of Gynecology and Obstetrics
GO: Gene ontology
KEGG: Kyoto Enrichment of Genes and Genomes
NCI: National Cancer Institute.
NGS: next-generation sequencing.
OC: Ovarian cancer
OCCC: Ovarian clear cell carcinoma
OS: Overall survival
SNVs: single nucleotide variants
