# Supplementary material for: Next-generation sequencing-based genomic profiling analysis reveals novel mutations for clinical diagnosis in Chinese primary epithelial ovarian cancer patients
Source: J Ovarian Res. 2019 Feb 20;12:19. doi: 10.1186/s13048-019-0494-4 (PMC6381667; doi:10.1186/s13048-019-0494-4)

A

ID	Chr	Position	Ref	Alt	Mutation Annotation	Sanger Sequencing
YXZ	chr17	39324333	T	A	KRTAP4-3(NM_033187):c.A92T:p.Q31L	Seqeucencing failed
SMF	chr17	7578508	C	T	TP53(NM_001126115):c.G26A:p.C9Y	**Validated**
SMF	chr17	18682505	T	C	FBXW10(NM_001267586):c.T2894C:p.V965A	**Validated**
SMF	chr19	58385748	G	A	ZNF814(NM_001144989):c.C1010T:p.A337V	Seqeucencing failed
QLF	chr17	7577539	G	A	TP53(NM_001126115):c.C346T:p.R116W	**Validated**
YHA	chr17	7577120	C	T	TP53(NM_001126115):c.G422A:p.R141H	**Validated**
GMY	chr17	7578536	T	C	TP53(NM_001126118):c.A277G:p.K93E	**Validated**
DYM	chr17	7577121	G	A	TP53(NM_001126115):c.C421T:p.R141C	**Validated**
ZLF	chr17	7573991	C	A	TP53(NM_001126115):c.G640T:p.E214X	Seqeucencing failed
JXX	chr17	7577534	C	A	TP53(NM_001126115):c.G351T:p.R117S	Seqeucencing failed
LGZ	chr17	7578265	A	T	TP53(NM_001126115):c.T188A:p.I63N	**Validated**

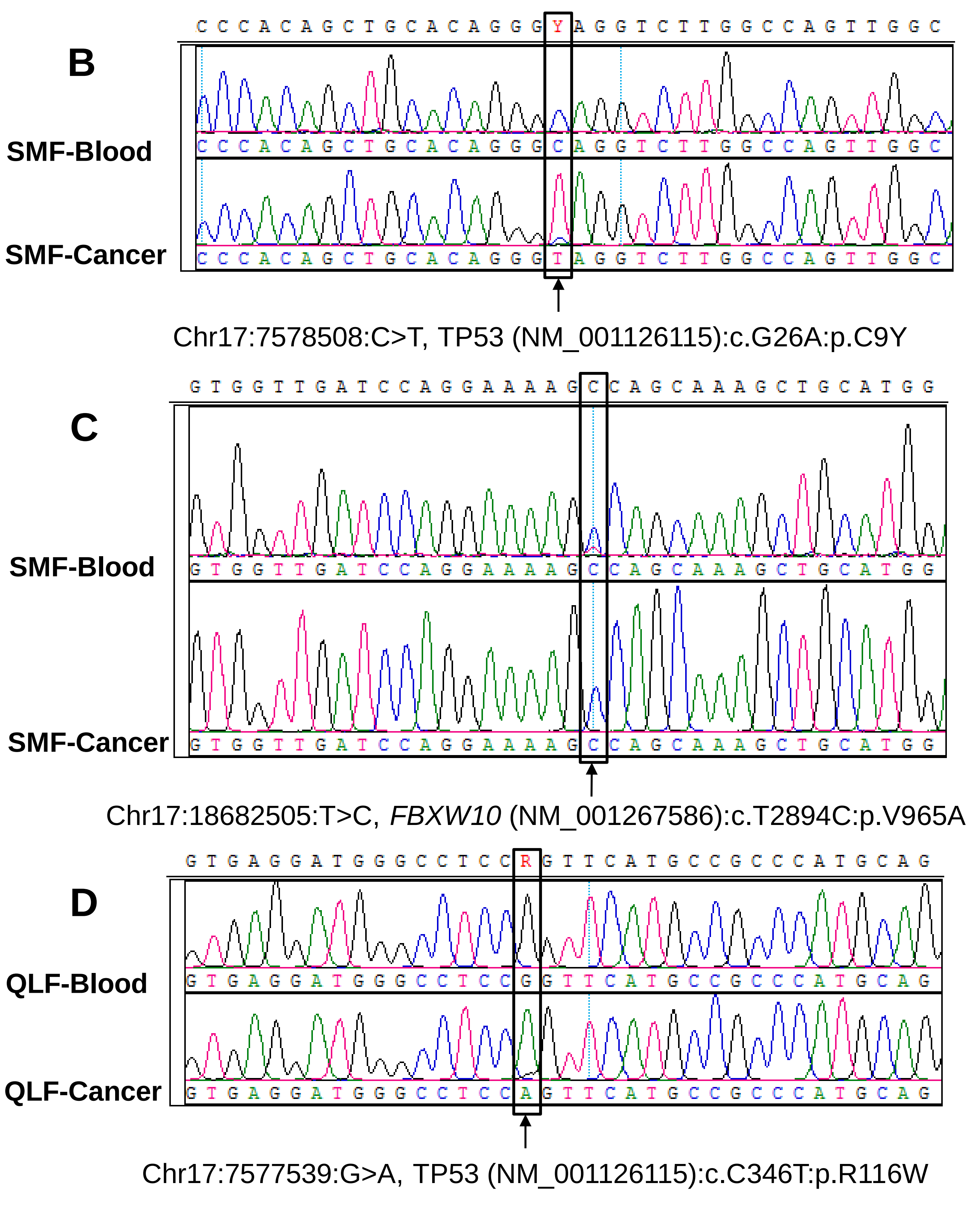

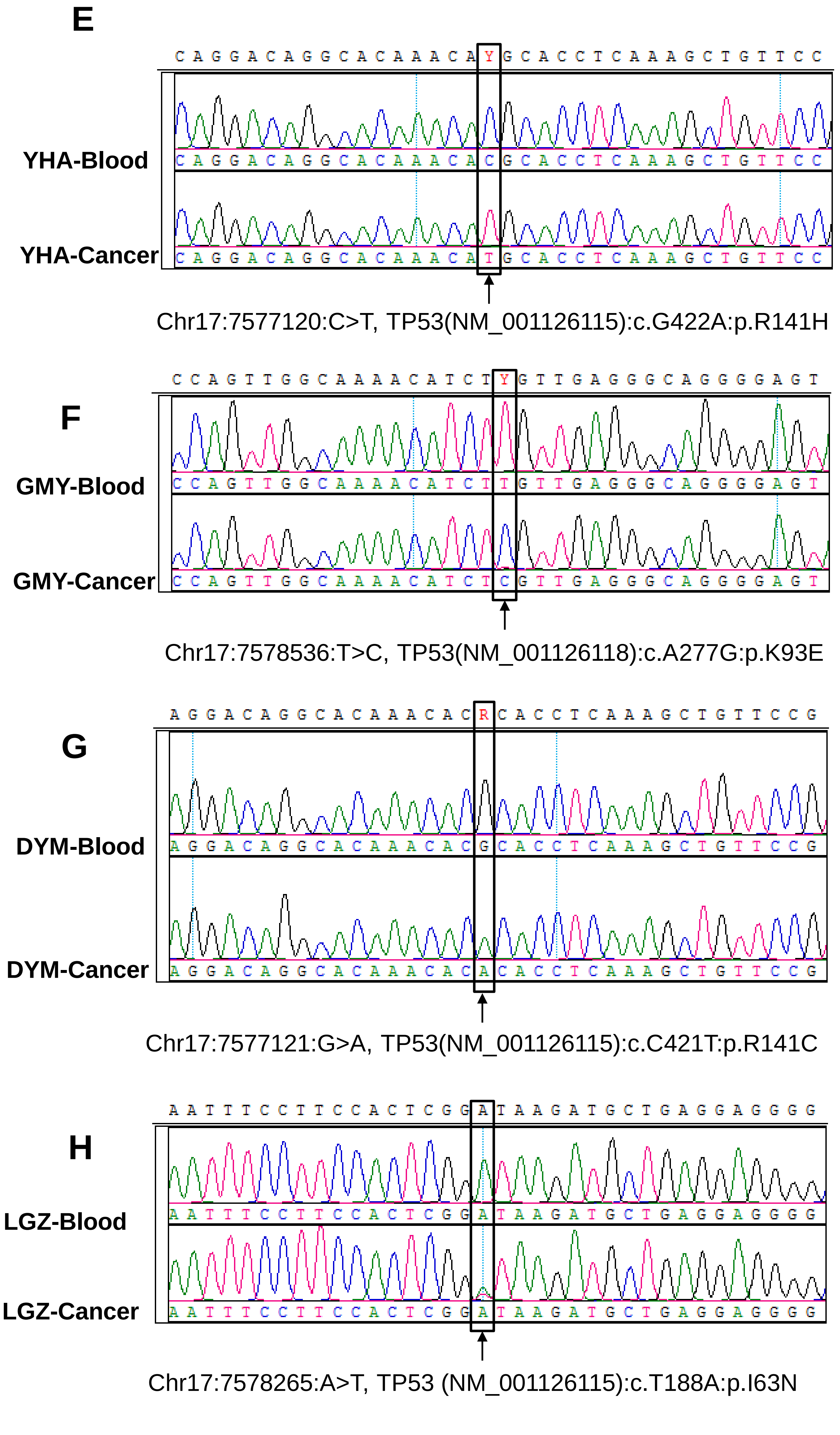

Supplement: Supplementary file 1 — Figure S1. Result of sanger sequencing. (A) Eleven mutations which were sanger sequenced. (B) - (H) Seven mutations which were validated to be true. (PDF 83 kb) [file 13048_2019_494_MOESM1_ESM.pdf]
