# Supplementary figures and images for: Next-generation sequencing-based genomic profiling analysis reveals novel mutations for clinical diagnosis in Chinese primary epithelial ovarian cancer patients
Source: J Ovarian Res. 2019 Feb 20;12:19. doi: 10.1186/s13048-019-0494-4 (PMC6381667; doi:10.1186/s13048-019-0494-4)

A

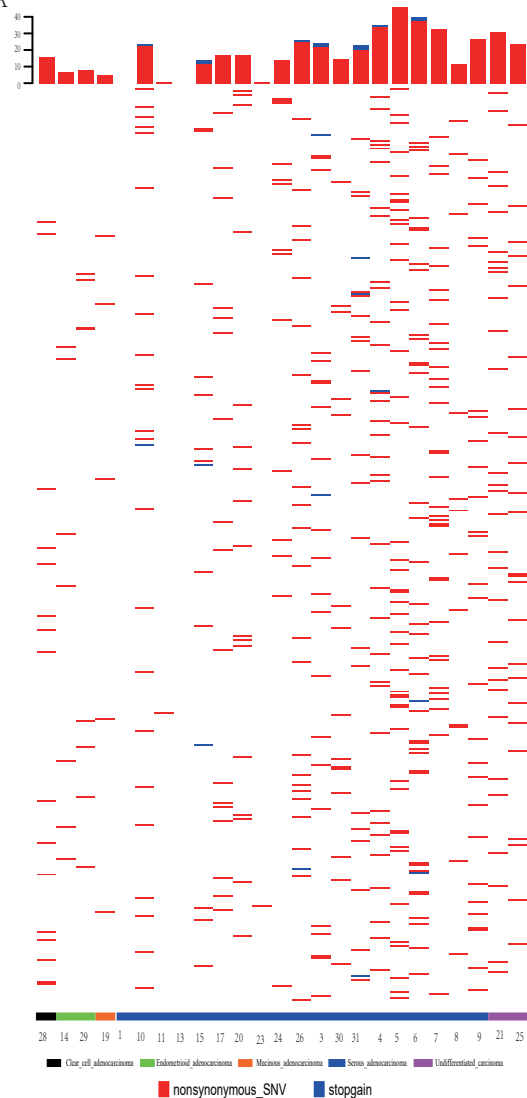

B

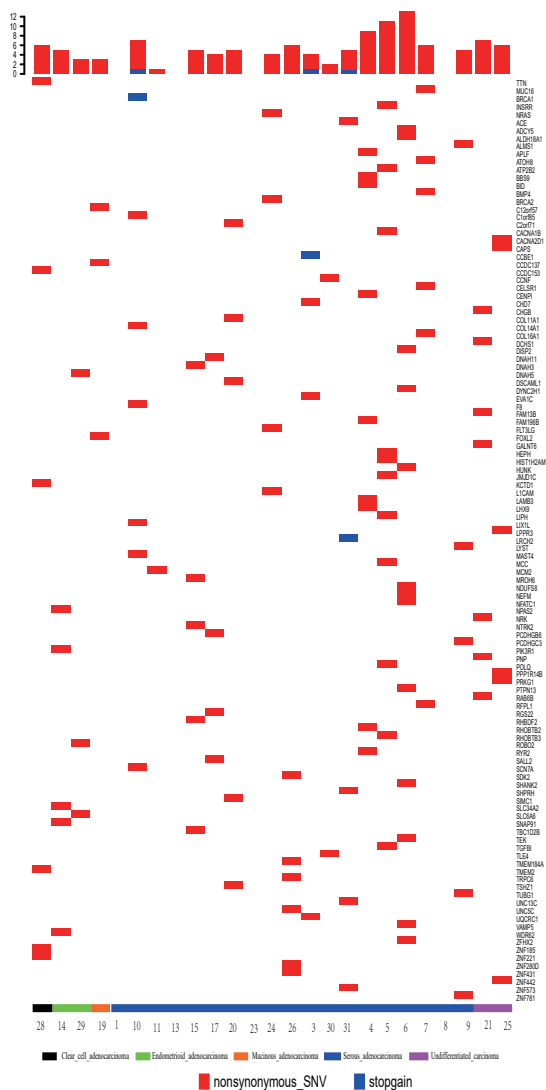

Supplement: Supplementary file 2 — Figure S2. Distribution of genes with novel mutations based on individual patients. (A) Distribution of 463 novel mutations in each patient with cancer. (B) Distribution of the 117 genes with novel mutations based on individual patients. (PDF 4100 kb) [file 13048_2019_494_MOESM2_ESM.pdf]

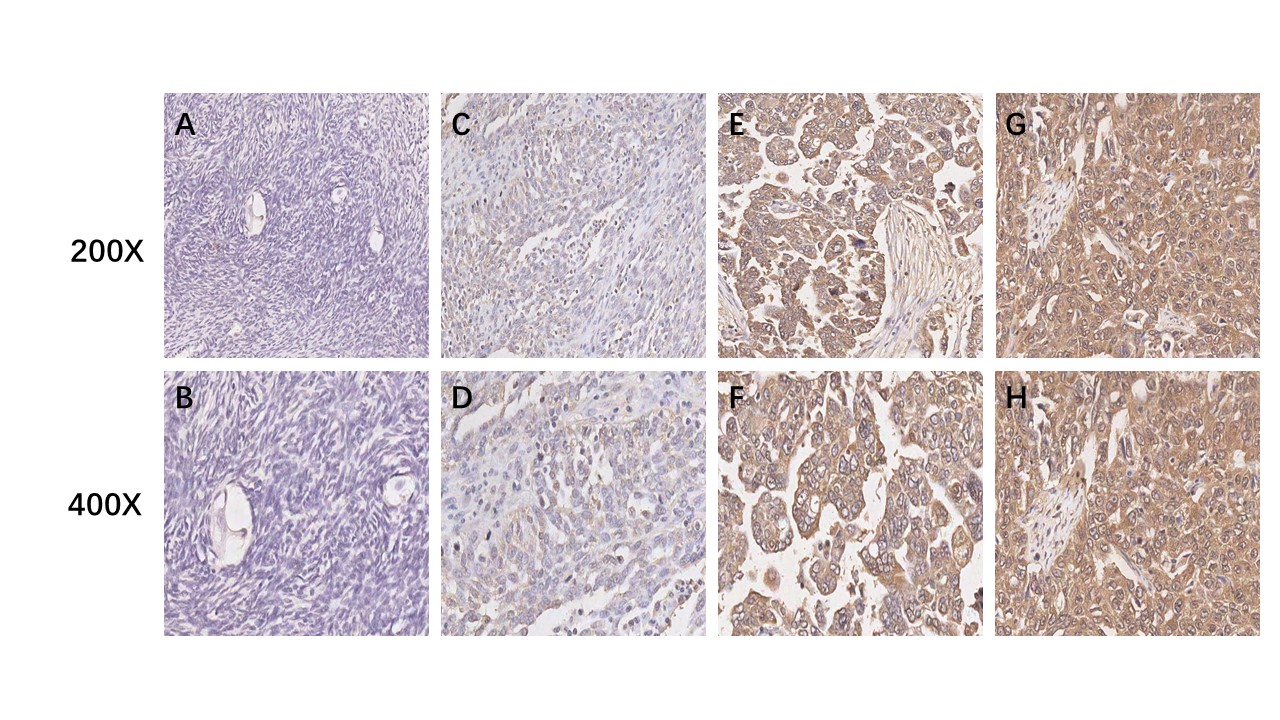

Supplement: Supplementary file 3 — Figure S3. Representative immunohistochemical staining for EPS8L1 expression. (A/B): Negative expression of EPS8L1 in normal ovarian tissue, 200×/400×. (C/D) Weakly positive expression of EPS8L1 in EOC tissue, 200×/400×. (E/F) Positive expression of EPS8L1 in EOC tissue, 200×/400×. (G/H) Strongly positive expression of EPS8L1 in EOC, 200×/400 ×. (JPG 303 kb) [file 13048_2019_494_MOESM3_ESM.jpg]

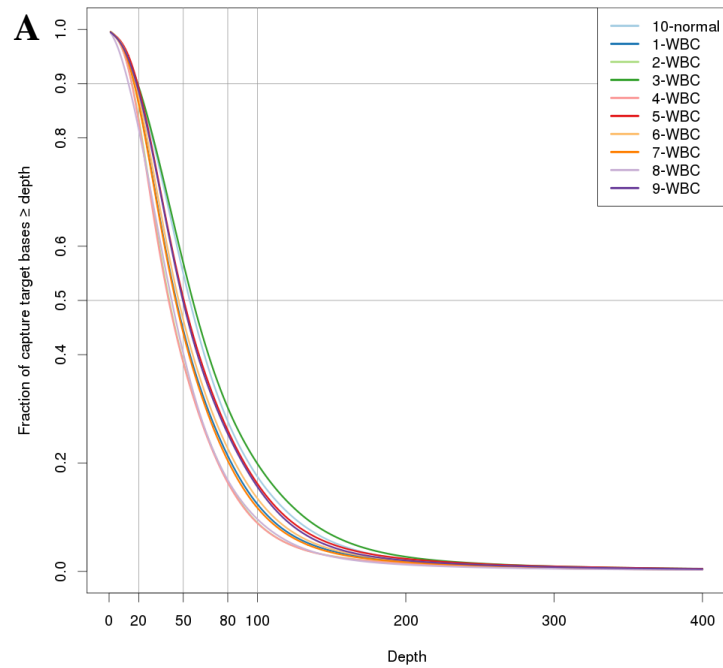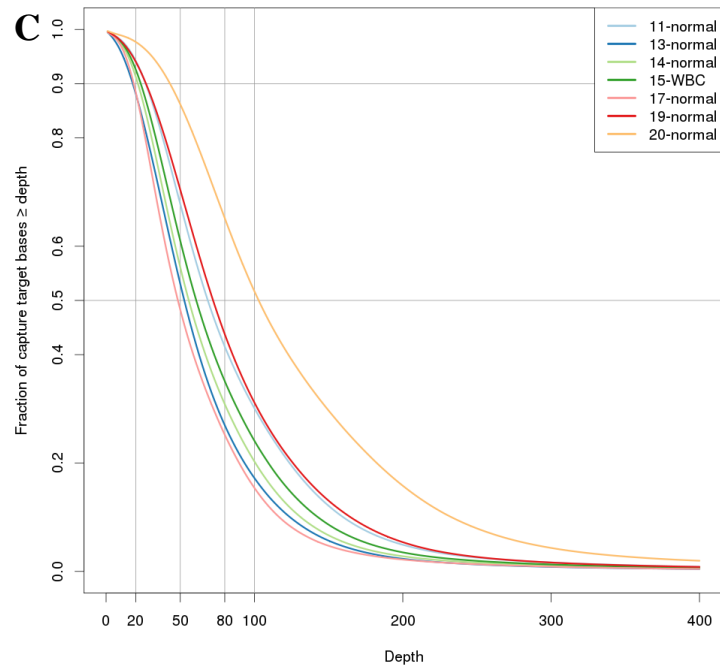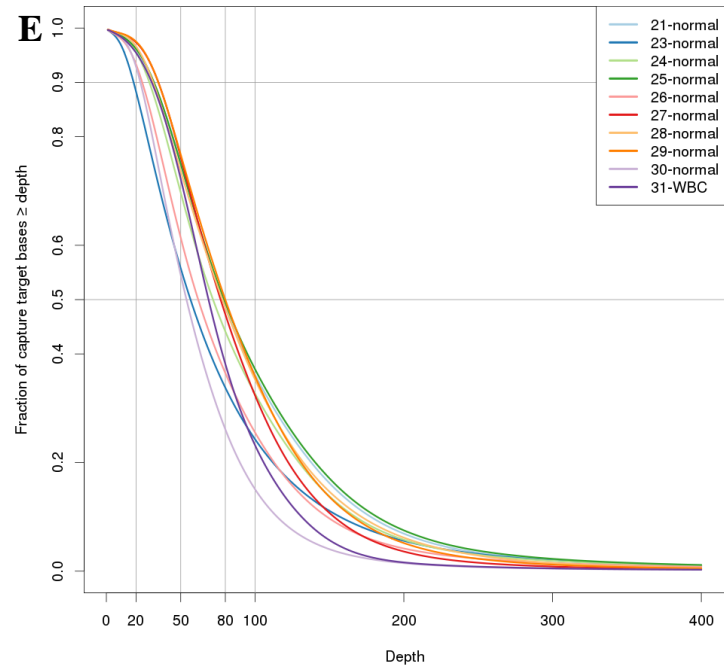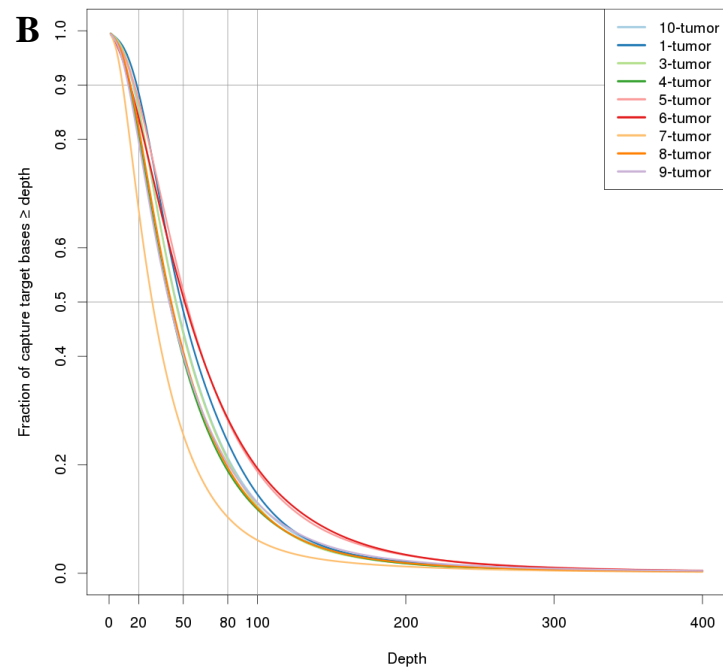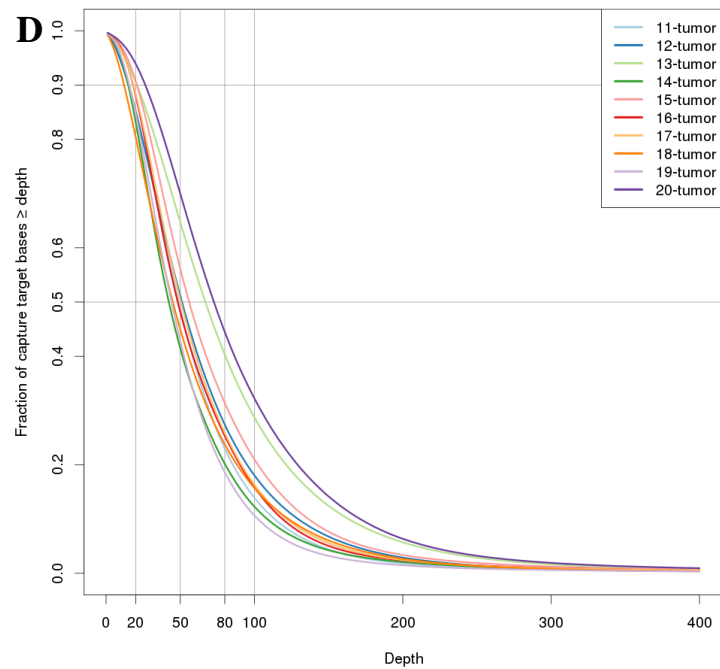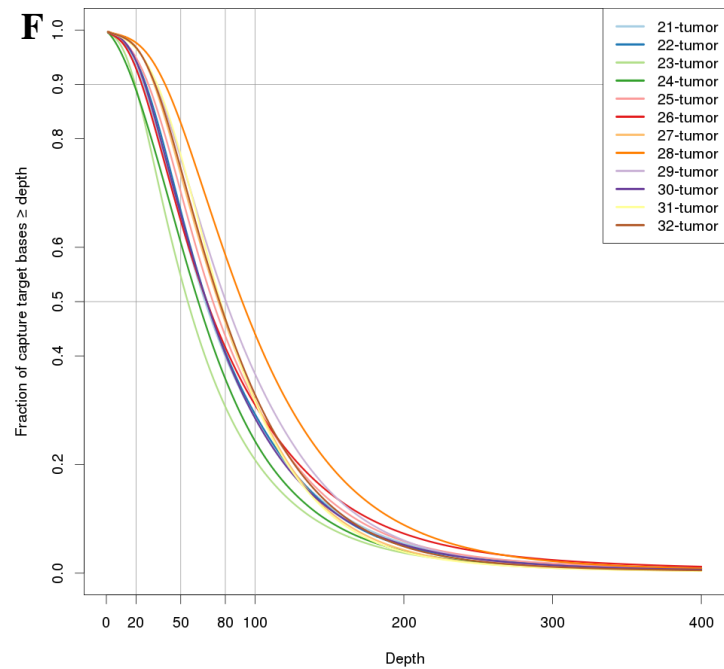

Supplement: Supplementary file 4 — Figure S4. Target region coverage. (A) Target region coverage of normal control group in sample 1–10. (B) Target region coverage of tumor group in sample 1–10. (C) Target region coverage of normal control group in sample 11–20. (D) Target region coverage of tumor group in sample 11–20. (E) Target region coverage of normal control group in sample 21–31. (F) Target region coverage of tumor group in sample 21–31. (PDF 632 kb) [file 13048_2019_494_MOESM4_ESM.pdf]
